# Antimycobacterial Activities of Endolysins Derived From a Mycobacteriophage, BTCU-1

**DOI:** 10.3390/molecules201019277

**Published:** 2015-10-22

**Authors:** Meng-Jiun Lai, Chih-Chin Liu, Shinn-Jong Jiang, Po-Chi Soo, Meng-Hsuan Tu, Jen-Jyh Lee, Ying-Huei Chen, Kai-Chih Chang

**Affiliations:** 1 Department of Laboratory Medicine and Biotechnology, Tzu Chi University, Hualien 97004, Taiwan; E-Mails: monjou@mail.tcu.edu.tw (M.-J.L.); pcsoo@mail.tcu.edu.tw (P.-C.S.); 101323102@stmail.tcu.edu.tw (M.-H.T.); 2 Department of Bioinformatics, Chung Hua University, Hsin-Chu City 97004, Taiwan; E-Mail: ccliu@chu.edu.tw; 3 Department of Biochemistry, Tzu Chi University, Hualien 97004, Taiwan; E-Mail: sjjiang@mail.tcu.edu.tw; 4 Department of Internal Medicine, Buddhist Tzu Chi General Hospital, Hualien 97004, Taiwan; E-Mails: e0139@tzuchi.com.tw (J.-J.L.); mt8731251@gmail.com (Y.-H.C.); 5 Department of Laboratory Medicine, Buddhist Tzu Chi General Hospital, Hualien 97004, Taiwan

**Keywords:** mycobacteriophage, endolysin, antimycobacterial

## Abstract

The high incidence of *Mycobacterium* infection, notably multidrug-resistant *M. tuberculosis* infection, has become a significant public health concern worldwide. In this study, we isolate and analyze a mycobacteriophage, BTCU-1, and a foundational study was performed to evaluate the antimycobacterial activity of BTCU-1 and its cloned lytic endolysins. Using *Mycobacterium smegmatis* as host, a mycobacteriophage, BTCU-1, was isolated from soil in eastern Taiwan. The electron microscopy images revealed that BTCU-1 displayed morphology resembling the Siphoviridae family. In the genome of BTCU-1, two putative lytic genes, BTCU-1_ORF7 and BTCU-1_ORF8 (termed *lysA* and *lysB*, respectively), were identified, and further subcloned and expressed in *Escherichia coli*. When applied exogenously, both LysA and LysB were active against *M. smegmatis* tested. Scanning electron microscopy revealed that LysA and LysB caused a remarkable modification of the cell shape of *M. smegmatis.* Intracellular bactericidal activity assay showed that treatment of *M. smegmatis*—infected RAW 264.7 macrophages with LysA or LysB resulted in a significant reduction in the number of viable intracellular bacilli. These results indicate that the endolysins derived from BTCU-1 have antimycobacterial activity, and suggest that they are good candidates for therapeutic/disinfectant agents to control mycobacterial infections.

## 1. Introduction

*Mycobacterium* *tuberculosis* (MTB) is the leading cause of tuberculosis which is a serious public health problem that results in millions of deaths around the world each year [1]. Approximately one-third of the world’s population is latently infected with MTB and at risk of reactivation [2]. The slow-growing MTB can persist in the latent state of asymptomatic infection for a long time, and the treatment of active diseases involves long multidrug regimens. Unfortunately, the world is at present struggling with multi-drug resistant (MDR) as well as extensively drug resistant (XDR) forms of MTB which threaten to make both the first and second line drugs ineffective [3,4]. The prevention of the spread of MTB is a continuing challenge, and finding novel agents to treat MDR and XDR MTB infections has become a priority.

Bacteriophages (phages) are viruses that infect and replicate within their bacterial hosts. The lytic phages have developed various ways to interfere with and kill their host cells. Therefore, phage therapy is being considered as a possible therapeutic alternative for the treatment of infections caused by MDR strains [5]. Particular attention has been paid to recombinant phage endolysins because of their potential to digest bacterial cell walls when applied exogenously, enabling their use as alternative antibacterials [6,7]. Presently, scientists have successfully tested the use of endolysins to control antibiotic-resistant bacterial pathogens in animal models [8].

Like other bacteriophages, mycobacteriophages were considered as an alternative therapy for *Mycobacterium* infection control [9]. Additionally, mycobacteriophage-encoded lytic endolysins have considerable potential to be effective antimicrobial agents-or enzybiotics-against a number of MDR and XDR MTB strains [10,11]. To date, several phages infecting *Mycobacterium* spp. have been reported [12,13,14]. Nonetheless, the isolation and genome description of mycobacteriophages in Taiwan still remain to be achieved.

In this study, we used *Mycobacterium smegmatis* as a rapidly growing host, and a mycobacteriophage was isolated from the soil near Buddhist Tzu Chi University and named BTCU-1. Electron microscopy images revealed morphology resembling the *Siphoviridae* family. The BTCU-1 genome is a linear double-stranded DNA of about 46 kb in length. To utilize mycobacteriophage lytic endolysins as therapeutic alternatives to antibiotics, we surveyed the genomic sequence of BTCU-1 and successfully identified two lytic-associated genes. Following cloning and expression/purification, various antimycobacterial activities of these two lytic proteins were determined *in vitro*.

## 2. Results and Discussion

### 2.1. Morphology of BTCU-1

**Figure 1:**
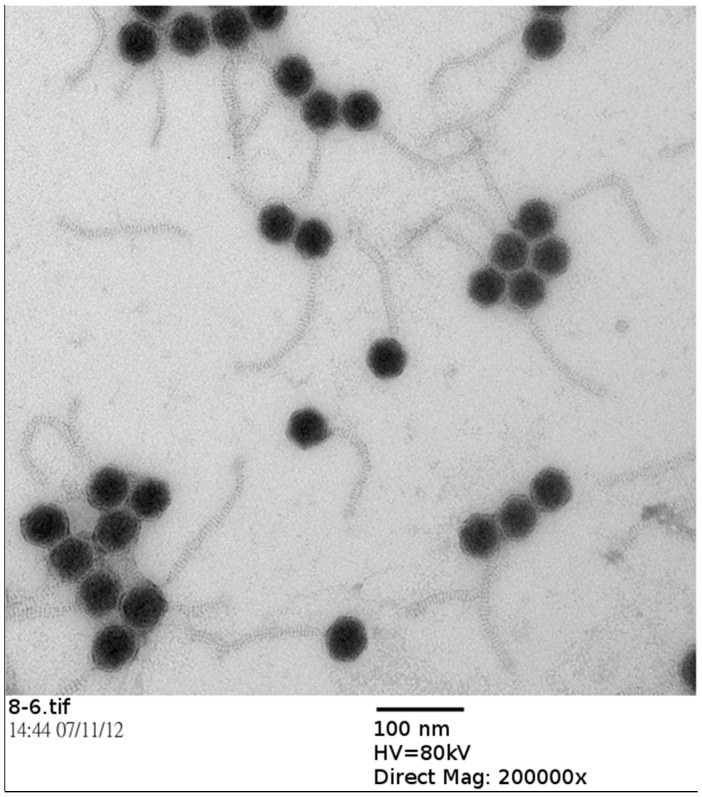
Electron micrograph of phage BTCU-1. Purified phage particles were negatively stained with 2% uranyl acetate. Bar 100 nm.

To classify mycobacteriophage BTCU-1 into a morphotype-specific group, the phage particles were examined by TEM. The electron microscopy images revealed that BTCU-1 possessed an icosahedral head (65 nm) and a non-contractile long tail (diameter, 12 nm; length, 180 nm), displaying morphology resembling the *Siphoviridae* family (Figure 1).

### 2.2. BTCU-1 Genome and Predicted Endolysins

The BTCU-1 genome is a linear double-stranded DNA of about 46 kb in length. The genomic sequence of BTCU-1 was deposited in GenBank (accession number KC172839). The complete genomic sequence of BTCU-1 showed more than 90% nucleotide identity to mycobacteriophages Rockstar and HelDan (Supplementary Figure S1). According to the functions predicted so far, in general, the structural and lysis proteins are encoded by one strand, and the proteins required for the maintenance of lysogeny are encoded by the opposite strand. One of the major differences of BTCU-1 from the other two genomes (Rockstar and HelDan) located at ORF4 and ORF5, with the limited knowledge that the product of ORF4 is a structural protein. In addition, about 2 Kb and 3.3 Kb extra genomic fragments were found after the repressor genes in the genomes of Rockstar and HelDan, respectively. Though the genome of BTCU-1 is suggested to contain genes related to lysogeny, the deletion of the genomic fragment in BTCU-1 may inactivate the repressor gene, causing the defect in establishing a lysogenic state. This presumption is supported by the clear plaques produced by BTCU-1 on *M. smegmatis*.

**Figure 2:**
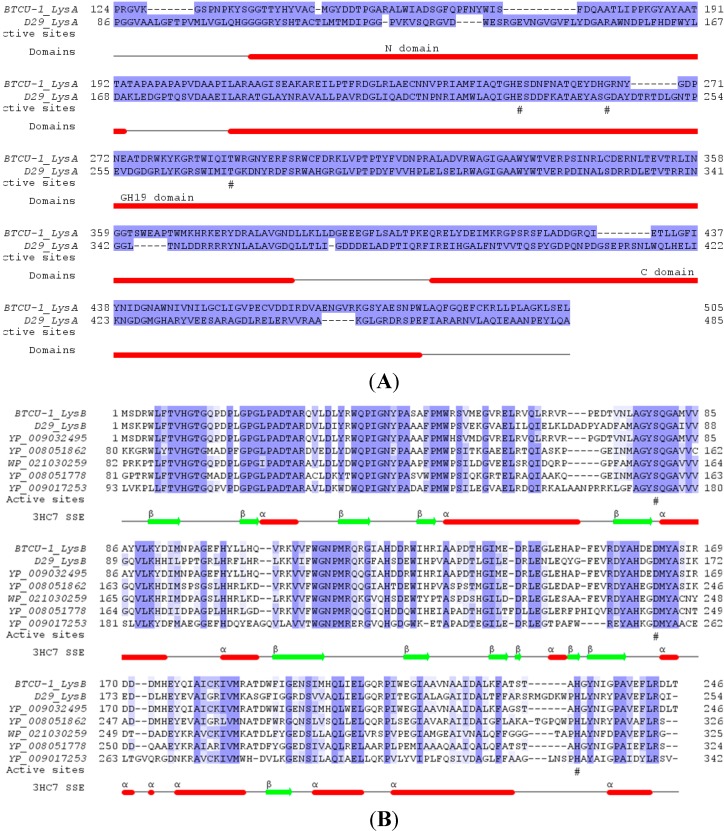
Multiple sequence alignment of BTCU-1 lytic associated proteins, LysA and LysB. (**A**) Sequence alignment between BTCU-1 LysA and D29 LysA (gp10, NP_046825). The modular organization of LysA is composed of an N-terminal domain with putative peptidase activity, a central catalytic GH19 (glycoside hydrolase family 19) domain, and a C-terminal domain possibly responsible for cell wall binding. The predicted catalytic residues in GH19 domains are marked by hash signs under the alignment; (**B**) Multiple sequence alignment of BTCU-1 LysB and its homologues, including D29 LysB (gp12, NP_046827) whose structure has been resolved (PDB code 3HC7). The predicted residues involving in catalysis (Ser82-Asp166-His240 in 3HC7) are marked by hash signs under the alignment. 3HC7 SSE, resolved secondary structural elements for D29 LysB; ribbons indicate helices and arrows indicate strands.

To utilize lytic endolysins as therapeutic alternatives to antibiotics, we surveyed the genomic sequence of BTCU-1 and successfully identified two lytic associated genes, BTCU-1_ORF7 and BTCU-1_ORF8 (termed *lysA* and *lysB*, respectively). LysA is a putative endolysin responsible for the cleavage of the peptidoglycan in the cell wall of mycobacteria. Sequence analysis suggests that LysA contains three domains: the N-terminal peptidase domain, the central catalytic GH19 (glycoside hydrolase family 19) domain, and the C-terminal cell wall binding domain (Figure 2A). LysB shares about 63% identity with the previously characterized LysB (gp12) in mycobacteriophage D29, and similarly lacks a possible peptidoglycan-binding domain at the N-terminal, while it presents in many D29 LysB homologues (Figure 2B) [15]. D29 LysB has been proved to be a mycolylarabinogalactan esterase required for the completion of lysis of the host mycobacterial cells [15]. It cleaves the ester linkage joining the mycolic acid-rich outer membrane to arabinogalactan, releasing free mycolic acids.

### 2.3. Cloning, Expression and Purification of LysA and LysB

**Figure 3:**
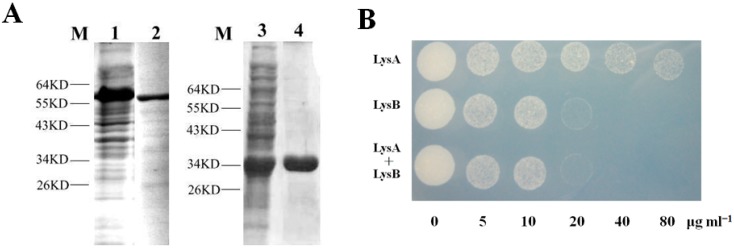
SDS-PAGE of purified LysA and LysB and their antimycobacterial activity. (**A**) Purified LysA and LysB were electrophoresed on a 12% SDS gel, followed by staining with Coomassie brilliant blue R-250. Lane M, molecular weight markers; lane 2, purified LysA; lane 4, purified LysB; Lanes 1 and 3 indicate crude extract of individual encoding strains about four hours after induction; (**B**) The minimal bactericidal concentration (MBC) of LysA was over 80 μg·mL^−1^, and the MBC of LysB was between 20 to 40 μg·mL^−1^.

To confirm that LysA and LysB are mycobacteriophage lytic associated enzymes, their genes (BTCU-1_ORF7 and BTCU-1_ORF8) from the BTCU-1 genome were cloned for further characterization. After the PCR reaction, two respective amplified sequences from the BTCU-1 genome were inserted into the *E. coli* expression vector pET-30b, yielding pET30b-LysA and pET30b-LysB, respectively. In *E. coli* BL21 (DE3), pET30b-LysA directed the synthesis of a single individual protein with an apparent molecular mass of about 63 kDa on a sodium dodecyl sulfate polyacrylamide gel electrophoresis (SDS-PAGE) (Figure 3A). Similarly, pET30b-LysB directed the synthesis of a single individual protein with an apparent molecular mass of about 34 kDa on an SDS gel (Figure 3A).

### 2.4. Antimycobacterial Activity of BTCU-1 and Purified LysA and LysB

We first tested the lytic activity of BTCU-1 against two *M. smegmatis* strains and seven *M. tuberculosis* isolates listed in Table 1. BTCU-1 showed broad lytic host range, affecting almost all the tested *Mycobacterium* strains except TCGH59490 (Supplementary Figure S2). To study the antibacterial activity of LysA and LysB, *M. smegmatis* ATCC 14468 was examined for its susceptibility to these proteins. As shown in Figure 3B, the MBC of LysA was over 80 μg·mL^−1^, and the MBC of LysB was between 20 and 40 μg·mL^−1^. To further analyze the antibacterial spectra of LysA and LysB, another three Gram-negative (*E. coli* ATCC 25922; *Acinetobacter baumannii* ATCC 17978 and *Salmonella enteric* BCRC 10746) and two Gram-positive bacteria (*Bacillus subtilis* BCRC 10447 and *Staphylococcus aureus* ATCC 25923) were used for the MBC assay. The MBC of LysA and LysB towards these five bacteria were higher than 200 μg·mL^−1^. We noticed that LysA and LysB had narrow spectra of antimicrobial activity against only *Mycobacteria* spp., with relatively lower bactericidal activity against other non-*Mycobacteria* species.

### 2.5. Electron Microscopy Experiments

**Figure 4:**
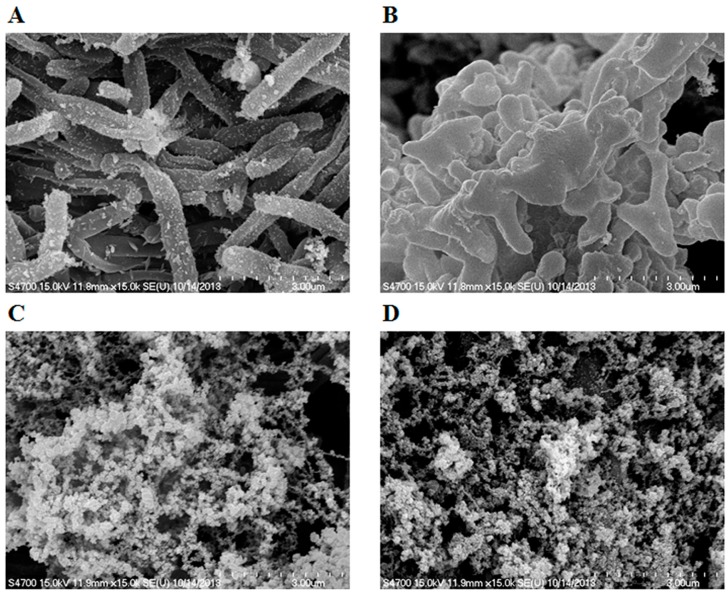
Scanning electron microscopy of *M. smegmatis* treated with LysA or LysB*.* (**A**) Untreated *M. smegmatis* cells; (**B**) *M. smegmatis* treated with 100 μg·mL^−1^ LysA for 24 h; (**C**) *M. smegmatis* treated with 100 μg·mL^−1^ LysB for 24 h; (**D**) *M. smegmatis* treated with 100 μg·mL^−1^ LysA combined LysB for 24 h.

The effects of LysA and LysB on the morphology of *M. smegmatis* were visualized using scanning electron microscopy (SEM). The exposure of bacteria to 100 μg·mL^−1^ LysA or LysB for 24 hour caused a remarkable modification of their cell shape, as shown by SEM. Untreated bacteria displayed a rough bright surface with no obvious cellular debris (Figure 4A). The crinkled and irregular spheroidal cell morphology was observed in the cells of *M. smegmatis* after exposed to LysA as shown in Figure 4B. The treatment with LysB resulted in notable bacterial rupture and breakdown in *M. smegmatis* (Figure 4C), and treatment with LysA combined LysB exhibited a wide range of significant abnormalities, including the collapse of the cell structure and notable cracked cells in *M. smegmatis* (Figure 4D).

### 2.6. LysA and LysB Kill Intracellular M. smegmatis

**Figure 5:**
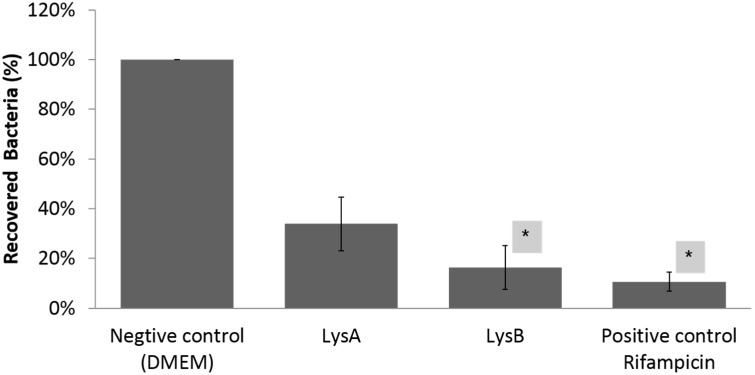
The intracellular survival of *M. smegmatis* after 12 h treated with LysA or LysB. Survival was expressed as the percentage in the recovered number of viable intracellular *M. smegmatis* compared with the negative control (incubation with DMEM). The differences in percentage in the recovered number between treated with LysA or LysB and negative control were analyzed by the Student’s *t*-test. Each bar represents the average value of three independent experiments, and the error bars represent the standard deviations. * *p* < 0.05.

To determine the effect of BTCU-1 derived endolysins on intracellular *M. smegmatis,* RAW 264.7 macrophage cell lines were infected with *M. smegmatis* ATCC 14468, and 2 h after infection, treated with LysA or LysB for 12 h. As shown in Figure 5, the viabilities of intracellular *M. smegmatis* dropped to <40% following incubation with LysA compared to the negative control (incubation with DMEM). However, the viabilities of *M. smegmatis* dropped to <20% following incubation with LysB for the same period of time. These results suggest that BTCU-1 derived endolysins can effectively decrease the number of intracellular *M. smegmatis*.

### 2.7. Discussion

The prevention of the spread of MTB is a continuing challenge, and finding novel agents to treat MDR and XDR MTB infections has become a priority. Mycobacteriophages own the abilities to attack and kill mycobacteria and can potentially serve as an alternative option to combat mycobacteria infection.

In this work, a mycobacteriophage was isolated from the soil near Buddhist Tzu Chi University, Taiwan and was named BTCU-1. To our knowledge, this is the first detailed study of a mycobacteriophage isolated from Taiwan. Similar to other mycobacteriophages reported, BTCU-1 showed a broad host range, being able to lyse almost all the tested *Mycobacterium* strains. This suggests its use as an alternative sanitation or disinfectant agent will be highly feasible for application in controlling MTB infections [16]. In order to have a better understanding about mycobacteriophage distribution in Taiwan, more work on the isolation and characterization of mycobacteriophages is still needed.

*Mycobacterium* spp. possess unusual cell wall core, which consists of a peptidoglycan layer covalently attached to a mycolic acid layer via the polysaccharide arabinogalactan [17]. Upon infection, mycobacteriophages produce lysins to digest the peptidoglycan and mycolic acid layers of the host cell wall, causing the rupture of the bacterial cells and the release of the phage particles. The ability to lyse mycobacterial cells makes lysins extremely important. There have been hundreds of mycobacteriophage genomes sequenced [18], and the sequence features of the major types of lysins have been analyzed [11,15]. Nevertheless, the evaluation of their antimycobacterial effect, particularly applied exogenously, is still very limited. In the example of mycobacteriophage Ms6, its LysA was characterized as a peptidoglycan amidase; however, in culture, it showed no antibacterial effect on either *M. tuberculosis* or *M. smegmatis* [19].

In this study, we surveyed the genomic sequence of BTCU-1 and identified two lytic associated genes, BTCU-1_ORF7 and BTCU-1_ORF8 (termed *lysA* and *lysB*, respectively). Sequence analysis suggests that LysA is a putative endolysin responsible for the cleavage of the peptidoglycan in the cell wall of mycobacteria, whereas LysB probably acts at the mycolylarabinogalactan bond to liberate free mycolic acid. To our surprise, either LysA or LysB alone, while applied exogenously, could cause a remarkable modification of the cell shape of *M. smegmatis* and also cause cell death. Scanning electron microscopy (SEM) result showed that LysA-treated *M. smegmatis* displayed crinkled and irregular spheroidal morphology. A simple explanation for this is that the peptidoglycan in the cell wall of mycobacteria was partially removed by the action of LysA, and the membrane tension caused the cell to form a characteristic spherical shape. However, how the exogenous LysA permeates through the mycolic acid-rich outer membrane to reach the substrates remains to be discovered. Furthermore, *in vitro* and *in vivo* antimicrobial assays showed that LysB possessed better bactericidal capacity than LysA while applied exogenously. Based on the results of SEM and sequence analysis, we can only presume that LysB proteins reach their host mycolylarabinogalactan targets from the outside, and decrease the integrity of the mycolic acid linkage to the arabinogalactan-peptidoglycan layer. This may directly affect the cell wall permeability barrier in mycobacteria. However, the mechanism of LysB that ultimately results in the bacteriolysis seen in these experiments is a question worthy of investigation.

## 3. Experimental Section

### 3.1. Bacteria, Vector and Growth Conditions

The bacterial strains and plasmids used in this study are listed in Table 1. *M. smegmatis* strains were grown at 37 °C in Middlebrook 7H9 medium (Difco, Detroit, MI, USA) with shaking, or on Middlebrook 7H11 agar (Difco) supplemented with 0.5% glycerol. *E. coli* strains were grown at 37 °C in Luria-Bertani (LB) broth or agar supplemented with 100 μg·mL^−1^ ampicillin or 50 μg·mL^−1^ kanamycin, when appropriate.

### 3.2. Isolation and Characterization of the Mycobacteriophage

In this study, *M. smegmatis* ATCC 14468 is a non-virulent mycobacterial strain and was used as a host for phage isolation. The soil sample was collected from sites near Buddhist Tzu Chi University, Hualien, Taiwan. The land is owned by Tzu-Chi Foundation, the founder of Tzu Chi University, and no specific permission was required to access the land or collect the samples. No endangered or protected species were found or are registered in this area. Each time, about 300–500 g of soil was collected from a surface of 100 cm^2^, at a depth of 3–5 cm. We believe the environmental impact is minimal. The mycobacteriophage isolation and purification process was performed according to the procedures described by Pope *et al.* [12]. Phage morphology was examined by TEM of negatively stained preparations [20]. A drop of approximately 10^10^ PFU/mL was applied to the surface of a formvar-coated grid (200 mesh copper grids), negatively stained with 2% uranyl-acetate and then examined in a Hitachi H-7500 transmission electron microscope (Hitachi Company, Tokyo, Japan) operated at 80 kV.

**Table 1:** Bacterial strains, plasmids, and oligonucleotide primers used in this study.

Strain, Plasmid, or Primer	Relevant Characteristics, Description, or Sequence	Source
**Bacterial Strains**		
*M. smegmatis* ATCC 14468	reference strain	American Type Culture Collection (ATCC)
*M. smegmatis* mc^2^155 (ATCC 70084)	reference strain	ATCC
*M. tuberculosis* TCGH 57612	Clinical strain	Buddhist Tzu Chi General Hospital (BTCGH)
*M. tuberculosis* TCGH 58339	Clinical strain	BTCGH
*M. tuberculosis* TCGH 58821	Clinical strain	BTCGH
*M. tuberculosis* TCGH 59490	Clinical strain	BTCGH
*M. tuberculosis* TCGH 62046	Clinical strain	BTCGH
*M. tuberculosis* TCGH 45545	MDR Clinical strain	BTCGH
*M. tuberculosis* TCGH 17037	XDR Clinical strain	BTCGH
*E. coli* Top10	Laboratory strain for TA cloning use	Invitrogen, San Diego, CA, USA
*E. coli* BL21 (DE3)	Laboratory strain for protein expression	Invitrogen
*E. coli* ATCC 25922	Gram-negative reference strain	ATCC
*A. baumannii* ATCC 17978	Gram-negative reference strain	ATCC
*S. enteric* BCRC 10746	Gram-negative reference strain	Bioresource Collection and Research Center (BCRC)
*B. subtilis* BCRC 10447	Gram-positive reference strain	BCRC
*S. aureus* ATCC 25923	Gram-positive reference strain	ATCC
**Plasmids**		
pGEM-T-easy	3015-bp *E. coli* vector, *Amp*^r^, *P*_lac_, *lac*Z	Promega, San Diego, CA, USA
pET30b	5421-bp *E. coli* vector, *Km*^r^, *P*_T7_, His-Tag	Novagen, Madison, WI, USA
pGEM-LysA	pGEM-T-easy::1566 bp of *lysA*	This study
pGEM-LysB	pGEM-T-easy::741 bp of *lysB*	This study
pET30b-LysA	pET-30b::1566 bp of *lysA*; His-Tag-LysA under *P*_T7_	This study
pET30b-LysB	pET-30b:: 741 bp of *lysB*; His-Tag-LysB under *P*_T7_	This study
**Primers**		
BTCU-1_*lysA*-FP	5ʹ-ATGACGGAACGGGTACTCCC-3ʹ	This study
BTCU-1_*lysA*-RP	5ʹ-TCACTGCTCGATGACCCTGTT-3ʹ	This study
BTCU-1_*lysB*-FP	5'-GTGAGCGACCGCTGGCT-3ʹ	This study
BTCU-1_*lysB*-RP	5ʹ-TCATGTCAAGTCGCGTAGAAACT-3ʹ	This study

### 3.3. Phage DNA Preparation and Genome Sequencing

Phage DNA isolation was performed as previously described in detail [21]. The genome of mycobacteriophage was sequenced using the Ion Torrent PGM 314 chip (approximately 500 × coverage rate, Thermo Fisher Scientific, Waltham, MA, USA). The order of assembled contigs was predicted from the genomic sequences of Mycobacteriophage Rockstar (GenBank accession number JF704111), and Mycobacteriophage HelDan (accession no. JF957058) and confirmed by PCR. Primer walking was used to fill the gaps. Gene prediction was performed using GenMark.hmm [22], GenMarkS and Glimmer [23], followed by a manual correction where needed. All genes were annotated by BLAST searches of the GenBank databases. tRNA genes were predicted using the tRNAscan-SE tools [24].

### 3.4. Cloning/Purification of Endolysins

Genomic DNA of the mycobacteriophage BTCU-1 was extracted as previously described in detail [21]. Plasmid DNA was purified from *E. coli* using a QIAprep Spin MiniPrep Kit (Qiagen, Hilden, Germany). Polymerase chain reaction (PCR) amplification, with the DNA of BTCU-1 as a template, was carried out to produce two putative endolysin genes *lysA* and *lysB*. To construct the LysA expression vector, a 1566-base pair DNA fragment of *lysA* was amplified by PCR with BTCU-1_*lysA*-FP and BTCU-1_*lysA*-RP primers and cloned into a TA cloning site of pGEM-T-easy (Promega); the resulting recombinant DNA (BTCU-1_*lysA*) was digested with *Eco*RI and cloned into the *Eco*RI sites of pET-30b (Novagen). The resulting plasmid, pET30b-LysA, was then used to transform *E. coli* strain BL21 (DE3) for expression. The pET30b-LysB expression vector was constructed in a similar approach to pET30b-LysA, using BTCU-1_*lysB*-FP and BTCU-1_*lysB*-RP as primers. All of the constructed plasmids for expression were confirmed by DNA sequencing. The subsequent expression and purification procedures of the recombinant proteins were carried out according to the processes as described by Lai *et al.* [21]. The *E. coli* BL21 (DE3) strains, harboring the plasmids pET30b-LysA or pET30b-LysB were grown overnight in LB medium containing kanamycin (50 μg/mL). The overnight culture of the transformed *E. coli* was diluted 100 times with the same medium and incubated at 37 °C with shaking (150 rpm) until the optical density of the medium at OD_600nm_ reached 0.5. The expression of the target gene was induced by the addition of isopropyl-l-d-thiogalacto pyranoside at a final concentration of 0.1 mM. After further incubation for 3 h, the cells were harvested by centrifugation. The cell pellet was suspended in 10 mL of lysisequilibration-wash (LEW) buffer containing 50 mM NaH_2_PO_4_/300 mM NaCl (pH 8.0), disrupted by sonication and centrifuged at 10,000 *g* for 15 min to remove debris. Crude supernatant was loaded onto Protino Ni-TED packed columns (MACHEREY-NAGEL, Düren, Germany) equilibrated with LEW buffer. The fractions were eluted with the elution buffer containing 50 mM NaH_2_PO_4_/300 mM NaCl/250 mM imidazole (pH 8.0). Active fractions were pooled and dialyzed against the elution buffer and concentrated by Amicon Ultra-0.5 centrifugal filter (MILLPORE, Bedford, MA, USA). The concentration of each purified protein was determined by the Bradford assay using bovine serum albumin as a standard.

### 3.5. Antimycobacterial Assays

The host-range of BTCU-1 was determined by the plaque-forming method, adapting a spot-test technique described by Rybniker *et al.* [25], with some modifications as detailed below. BTCU-1 was prepared in a phage buffer (10 mM Tris, pH 7.5, 1 mM MgSO_4_, and 70 mM NaCl) and diluted to 10^6^ PFU/mL. The mycobacterial strains used in this test were grown overnight in Middlebrook 7H9 broth (Difco) (for *M. smegmatis*) or grown on 7H11 agar (for 7 *M. tuberculosis* clinical strains) at 37 °C. Until the bacteria grew to an appropriate concentration, the mycobacterial strains were diluted to an OD_600_ of 0.1. Two hundred microliters (µL) of mycobacteria were mixed with 200 µL of prepared BTCU-1 and added to 3 mL top agar containing 1 mM CaCl_2_ and poured onto 7H11 agar plates. Plates were incubated at 37 °C for four days for the fast-growing *Mycobacteria*, and for up to 4–6 weeks for the slow-growing strains.

*In vitro* antimicrobial assays were performed for the purified LysA and LysB. Antimicrobial activity was determined as described by Chen *et al.* [26], with some modifications as detailed below. Bacteria were grown overnight in Middlebrook 7H9 medium or Mueller–Hinton broth (Difco) at 37 °C, and during the mid-logarithmic phase, bacteria were diluted to 10^6^ colony*-*forming units (CFU)/mL in phosphate buffer. LysA and LysB were serially diluted in the same buffer to the concentration range from 10 to 400 μg·mL^−1^. Fifty microliter (µL) of bacteria was mixed with fifty µL of LysA and LysB at varying concentrations followed by incubation at 37 °C for 24 h without shaking. At the end of incubation, bacteria were inoculated on Middlebrook 7H11 agar or Mueller-Hinton agar, and allowed growth at 37 °C for four days. The lowest concentration of LysA or LysB on the agar plate which displayed no bacterial growth is defined as the minimal bactericidal concentration (MBC). All experiments were performed in triplicate.

### 3.6. Scanning Electron Microscopy

The scanning electron microscopy images were prepared as previously described in detail [16]. *M. smegmatis* ATCC 14468 was grown in Middlebrook 7H9 medium to a log phase, harvested by centrifugation, washed twice with deionized water and resuspended in the same water. Approximately 10^8^ cells were incubated at 37 °C for 24 h with 100 μg·mL^−1^ LysA or LysB. Negative controls were run in the presence of phosphate buffer. The volume was adjusted to 100 µL. The treated cells were fixed with 2.5% (*w*/*v*) glutaraldehyde in 0.1 M cacodylate buffer and 1% tannic acid, thoroughly washed with the phosphate buffer and dehydrated with a graded ethanol series. After critical-point drying and gold coating, the samples were observed with a HITACHI S-4700 instrument (Hitachi Company, Tokyo, Japan) operated at 15 kV.

### 3.7. Intracellular Bactericidal Assay

Infection of macrophages and quantitation of intracellular bacteria were performed as already described with some modifications [27]. Mouse peritoneal macrophage cell line, RAW 264.7, was obtained from the American Tissue Culture Collection. Cells were cultured in Dulbecco’s Modified Eagle Medium (DMEM; Difco Laboratories) supplemented with 10% fetal bovine serum and 2 mM l-glutamine. For the assays described in this article, RAW 264.7 macrophages (10^6^) were treated with trypsin, washed, and seeded on a six-well tissue culture plate (Corning Costar, Cambridge, MA, USA) and allowed to grow overnight at 37 °C with an atmosphere of 5% CO_2_. *M. smegmatis* ATCC 14468 were used to infect RAW 264.7 macrophages. Bacteria were grown overnight in Middlebrook 7H9 medium at 37 °C, and during the mid-logarithmic phase, bacteria were diluted to 10^8^ CFU/mL in DMEM. Monolayers (∼10^6^ cells) were incubated with *M. smegmatis* at a ratio of 100 bacteria to 1 cell. Infection was allowed to occur for 2 h, and then the monolayers were washed with Hank’s Balanced Salt Solution (HBSS) twice and reincubated for two hours with medium containing kanamycin 50 µg·mL^−1^ to kill extracellular bacteria. After washed with HBSS three times, the *M. smegmatis*—infected monolayers were then incubated for 12 h with either 5 µg·mL^−1^ LysA and 5 µg·mL^−1^ LysB, and the control experiment was performed with DMEM or 5 µg·mL^−1^ Rifampicin. After the treatment of the phage-related endolysins, cell monolayers were washed three times and the medium was replaced with 1 mL of sterile distilled water to lyse the macrophages. After vigorous pipetting to ensure complete cell lysis, viable intracellular *M. smegmatis* were determined by quantitative plating of serial dilutions of the lysates on Middlebrook 7H11 agar. Each test was done three times in independent experiments, and the number of CFU recovered per well (mean number ± S.D.) was determined.

## 4. Conclusions

Our results indicate that BTCU-1 derived endolysins have antimycobacterial activity. These results also suggest that a variety of the genes encoding mycobacteriophage-related lytic endolysins can be readily isolated from the mycobacteriophage genomes. Use of these lytic endolysins as alternative sanitation or disinfectant agents will be highly feasible for application in controlling mycobacterium infections.

## Supplementary Materials

Supplementary materials can be accessed at: http://www.mdpi.com/1420-3049/20/10/19277/s1.
