# Supplementary material for: Antimycobacterial Activities of Endolysins Derived From a Mycobacteriophage, BTCU-1
Source: Molecules. 2015 Oct 22;20(10):19277–90. doi: 10.3390/molecules201019277 (PMC6332426; doi:10.3390/molecules201019277)
Supplement: Supplementary file 1 [file molecules-20-19277-s001.pdf]

Supplementary Materials

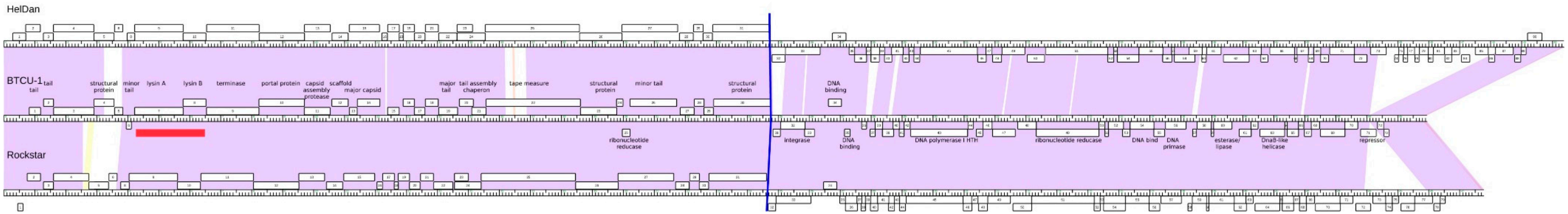

Figure S1. Genome maps of the three mycobacteriophages HelDan (JF957058), BTCU-1 (KC172839) and Rockstar (JF704111). The shading with colors from the violet towards the red end of the spectrum reflects a reduced level of similarity defined by BLASTN between the genomes. No shading means that there is no similarity with a BLASTN score of 10^{-4} or better. Key annotations of the gene products encoded by BTCU-1 are shown. The putative lysis cassette of BTCU-1 composed of *lysA* and *lysB* is highlighted by a red bar. The boundary between the two major gene clusters located on different strands is indicated by a blue line.

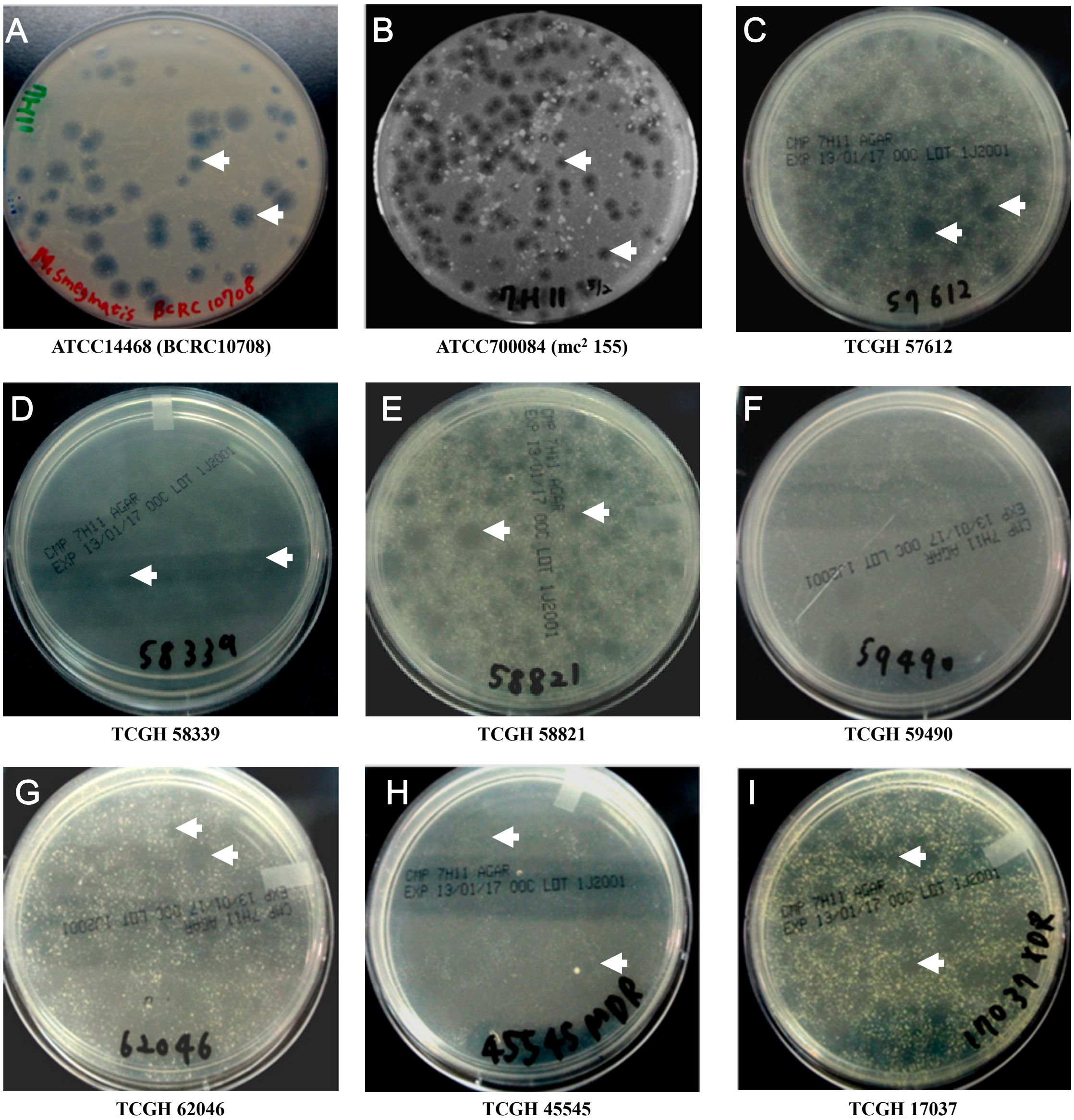

Figure S2. Lytic activity of BTCU-1 against two *M. smegmatis* strains and seven *M. tuberculosis* isolates was detected by the formation of clear plaques. The mixture of BTCU-1 with different *Mycobacterium* strains ((**A**,**B**) *M. smegmatis*; (**C**–**I**) *M. tuberculosis* clinical isolates) was added to agar and the plates were incubated. Some of the clear plaques formed are highlighted by white arrows. The lysis of the host bacteria *M. tuberculosis* TCGH 58339 (**D**) and 45545 (**H**) was very effective, producing clear plates.

In this result, BTCU-1 presented a broad lytic host range, affecting nearly all the tested *Mycobacterium* strains except *M. tuberculosis* TCGH59490 (F).
